# Histidine-rich glycoprotein inhibits TNF-α–induced tube formation in human vascular endothelial cells

**DOI:** 10.3389/fphar.2025.1561628

**Published:** 2025-03-21

**Authors:** Omer Faruk Hatipoglu, Takashi Nishinaka, Kursat Oguz Yaykasli, Shuji Mori, Masahiro Watanabe, Takao Toyomura, Masahiro Nishibori, Satoshi Hirohata, Hidenori Wake, Hideo Takahashi

**Affiliations:** ^1^ Department of Pharmacology, Kindai University Faculty of Medicine, Osakasayama, Japan; ^2^ Department of Internal Medicine 3—Rheumatology and Immunology, Friedrich-Alexander-University Erlangen-Nürnberg (FAU) and Universitätsklinikum Erlangen, Erlangen, Germany; ^3^ Department of Pharmacology, School of Pharmacy, Shujitsu University, Okayama, Japan; ^4^ Department of Translational Research and Dug Development, Graduate School of Medicine, Dentistry and Pharmaceutical Sciences, Okayama University, Okayama, Japan; ^5^ Department of Medical Technology, Graduate School of Health Sciences, Okayama University, Okayama, Japan

**Keywords:** histidine-rich glycoprotein, tumor necrosis factor-α, integrin, tube formation, angiogenesis, factor erythroid 2-related factor 2

## Abstract

**Introduction:**

Tumor necrosis factor-α (TNF-α)-induced angiogenesis plays a critical role in tumor progression and metastasis, making it an important therapeutic target in cancer treatment. Suppressing angiogenesis can effectively limit tumor growth and metastasis. However, despite advancements in understanding angiogenic pathways, effective strategies to inhibit TNF-α-mediated angiogenesis remain limited.

**Methods:**

This study investigates the antiangiogenic effects of histidine-rich glycoprotein (HRG), a multifunctional plasma protein with potent antiangiogenic properties, on TNF-α-stimulated human endothelial cells (EA.hy926). Tube formation assays were performed to assess angiogenesis, and gene/protein expression analyses were conducted to evaluate HRG’s effects on integrins αV and β8. The role of nuclear factor erythroid 2-related factor 2 (NRF2) in HRG-mediated antiangiogenic activity was also examined through nuclear translocation assays and NRF2 activation studies.

**Results:**

At physiological concentrations, HRG effectively suppressed TNF-α-induced tube formation in vitro and downregulated TNF-α-induced expression of integrins αV and β8 at both the mRNA and protein levels. HRG treatment promoted NRF2 nuclear translocation in a time-dependent manner. Furthermore, activation of NRF2 significantly reduced TNF-α-induced tube formation and integrin expression, suggesting that NRF2 plays a key role in HRG-mediated antiangiogenic effects.

**Discussion and Conclusion:**

Our findings indicate that HRG suppresses TNF-α-induced angiogenesis by promoting NRF2 nuclear translocation and transcriptional activation, which in turn inhibits integrin αV and β8 expression. Given the essential role of angiogenesis in tumor progression, HRG’s ability to regulate this process presents a promising therapeutic strategy for cancer treatment.

## 1 Introduction

Histidine-rich glycoprotein (HRG) is a 75 kDa plasma protein primarily synthesized in the liver and present in human plasma at physiological concentrations of approximately 63 μg/mL (IQR: 51.53–66.21 μg/mL) in healthy individuals (Koide et al., 1986; Kuroda et al., 2018). However, HRG levels decrease significantly under pathological conditions. In patients with systemic inflammatory response syndrome (SIRS), HRG levels are reduced to 28.72 μg/mL (IQR: 15.74–41.46 μg/mL), while in non-SIRS patients, the levels are slightly higher at 38.64 μg/mL (IQR: 30.26–51.81 μg/mL) (Kuroda et al., 2018). In sepsis patients, HRG levels drop even further to 8.71 μg/mL (IQR: 6.72–15.74 μg/mL) (Kuroda et al., 2018; Wake et al., 2016).

HRG modulates angiogenesis by affecting macrophage polarization and regulating key signaling mechanisms governing the migration and proliferation of endothelial cells (Rolny et al., 2011). The absence of HRG enhances platelet activation, accelerating the angiogenic switch (Ringvall et al., 2011). The antiangiogenic activity of HRG is primarily mediated by a 35-amino-acid fragment known as HRGP330, which disrupts focal adhesion in endothelial cells, inhibiting their motility and preventing vascular endothelial growth factor (VEGF)-induced angiogenesis (Dixelius et al., 2006). HRG also interacts with endothelial heparan sulfate in a Zn^2+^-dependent manner, blocking cell migration (Vanwildemeersch et al., 2006). *In vivo* studies have revealed a significant impact of HRG on tumor progression, with a >60% reduction in tumor growth reported in a mouse model (Olsson et al., 2004). These findings suggest that HRG is a potent inhibitor of tumor angiogenesis.

In contrast to the inhibitory role of HRG in angiogenesis, tumor necrosis factor-α (TNF-α) acts as a key promoter of this process. TNF-α is a key cytokine involved in processes such as angiogenesis, cell proliferation, and inflammation (Laha et al., 2021). It plays a crucial role in promoting angiogenesis, which is essential for metastasis and tumor growth (Charles et al., 2009; Oshima et al., 2014). TNF-α also performs significant functions in the early stages of cancer development, contributing to the tumor microenvironment and supporting the progression and survival of tumors (Zhou et al., 2017). TNF-α enhances the expression of various integrins on endothelial cells and promotes their activation; these integrins are important in mediating cell–extracellular matrix (ECM) interactions necessary for angiogenesis (Herzberg et al., 1996; Montecucco et al., 2008). These interactions promote the adhesion, migration, and survival of endothelial cells, which are key steps in the formation of new blood vessels (Aman and Margadant, 2023). Integrins are transmembrane receptors comprising α- and β-subunits, which mediate interactions between cells and the ECM (Barczyk et al., 2010). By facilitating processes such as cell adhesion, migration, and survival, integrins play a pivotal role in angiogenesis (Hood and Cheresh, 2002; Aman and Margadant, 2023). Activation of integrin in response to TNF-α signaling also highlights their potential as therapeutic targets. Blocking integrin–ECM interactions could provide a strategy to inhibit aberrant angiogenesis in diseases such as cancer (Pang et al., 2023). Beyond their role in cell adhesion, integrins also activate intracellular signaling pathways that regulate cellular responses to stress.

In these key pathways, nuclear factor erythroid 2-related factor 2 (NRF2) plays a vital role as a transcription factor regulating genes that respond to oxidative stress and inflammatory processes. NRF2 activation is essential for protecting endothelial cells from oxidative damage and maintaining vascular homeostasis (Ngo and Duennwald, 2022). Emerging evidence suggests that integrin-mediated signaling may influence NRF2 activity, linking cell adhesion mechanisms with oxidative stress responses. This interaction could be important in balancing angiogenesis with endothelial cell protection.

Although TNF-α has been shown to enhance the migration of endothelial cells, the detailed mechanisms underlying its role in tube formation remain unclear. This study was aimed at investigating the role of HRG in modulating TNF-α–induced integrin expression and tube formation in human endothelial cells. Additionally, we examined the involvement of NRF2, as we surmised its activation might be crucial in HRG-mediated inhibition of angiogenesis. The findings of this study shed light on mechanisms that may aid the development of therapeutic strategies to address abnormal angiogenesis in cancer and other pathological conditions.

## 2 Materials and methods

### 2.1 Reagents and antibodies

TNF-α was purchased from Gibco (PHC3011, Waltham, MA, United States). The integrin alpha V antibody was obtained from R&D Systems (AF1219-SP, Minneapolis, MN, United States), and anti-integrin beta eight antibodies were sourced from Thermo Fisher Scientific (PA5-100843, Waltham, MA, United States). The anti-integrin αVβ8 antibody was purchased from Sigma-Aldrich (ZRB1192, St. Louis, MO, United States), and the beta-actin antibody was obtained from Cell Signaling Technology (#3700, Danvers, MA, United States). The anti-Nrf2 antibody was obtained from Santa Cruz Biotechnology (sc-365949, CA, United States). Horseradish peroxidase-conjugated secondary antibodies, including anti-goat IgG from SeraCare Life Sciences, Inc. (5,220-0362, Milford, MA, United States), and rabbit anti-rabbit IgG and anti-mouse IgG from Cell Signaling Technology (#7076, #7074), were also used. Phalloidin-iFluor 555 Conjugate was sourced from Cayman Chemical (20,552, Ann Arbor, MI, United States). The NRF2 activator, bis-1,4-(4-methoxybenzenesulfonamidyl)naphthalene, was purchased from Merck (SML0959, Darmstadt, Germany). Other reagents were obtained from Nacalai Tesque (Kyoto, Japan) and were of the highest grade.

For RNA interference (RNAi) experiments, the following siRNAs were used: Negative Control siRNA from Bioneer (AccuTarget Negative control siRNA, SN-1001, Daejeon, Republic of Korea), and Integrin Beta 8 Silencer™ Select Pre-Designed siRNAs (IDs: s7598 and s7599) and Integrin Alpha V Silencer™ Select Validated siRNAs (IDs: s7570, s7569, and s7568) from Thermo Fisher Scientific.

### 2.2 Cell culture and treatment

EA.hy926 cells were sourced from American Type Culture Collection (Manassas, VA, United States). Dulbecco’s modified Eagle medium was supplied by Nissui Pharmaceutical (Tokyo, Japan). Human umbilical vein endothelial cells (HUVECs) and Endothelial Cell Growth Medium two were purchased from PromoCell (C-12200, C-22111, Heidelberg, Germany). The cells were maintained at 37°C in a humidified incubator with 5% CO_2_ until subconfluence was reached, after which they were seeded into 12-, 24-, or 96-well plates for further investigation.

### 2.3 Cell viability assay

The viability of cells was evaluated using the Cell Counting Kit-8 (Dojindo Laboratories, Kumamoto, Japan). A total of 1 × 10^4^ cells per 100 μL medium were plated into 96-well plates and exposed to TNF-α, HRG, and SML0959. The cells were incubated for 24 h at 37°C in a 5% CO_2_ humidified environment, after which the Cell Counting Kit-8 assay was performed. Following a 2-h reaction with the Cell Counting Kit-8 reagent, absorbance was measured at 450 nm using a microplate reader (Model 680, Bio-Rad Laboratories, Hercules, CA, United States). The viability of treated cells was expressed as a percentage compared to the control group.

### 2.4 Quantitative real-time polymerase chain reaction

Total RNA was isolated from cultured cells using TRI Reagent (Molecular Research Center, Cincinnati, OH, United States). RNA purity and concentration were determined using a NanoDrop One Spectrophotometer (Thermo Fisher Scientific). To synthesize cDNA, 2 μg of RNA was processed with the ReverTra Ace kit (Toyobo, Osaka, Japan). Real-time quantitative reverse transcription PCR (qRT-PCR) was carried out using the GeneAce SYBR kit (Nippon Gene, Tokyo, Japan) on a StepOnePlus Real-Time PCR System (Applied Biosystems, Foster City, CA, United States). The PCR conditions included an initial denaturation at 95°C for 30 s, followed by 40 cycles of 5 s at 95°C and 40 s at 60°C for annealing and elongation. Data analysis was conducted using the comparative 2^−ΔΔCT^ method, with *GAPDH* as the reference gene. Primer sequences used in this study are presented in Table 1.

**TABLE 1:** Primer Sequences Used for qPCR Analysis.

Gene name	Forward (5’-3’)	Reverse (5’-3’)
*integrin αV*	TAG​CAA​CTC​GGA​CTG​CAC​AAG​CTA	GGA​TTC​TCC​AGA​AGG​TGG​TTT​CG
*integrin β3*	CAG​ACA​CTC​CCA​CTT​GGC​ATC	TCC​TCA​GGA​AAG​GTC​CAA​TGT​G
*integrin β8*	CGA​AAC​CAA​GGT​TCT​GAG​CCC​A	CTT​GGA​TCT​CCA​CTG​AGG​CAG​T
*VEGF*	TTG​CCT​TGC​TGC​TCT​ACC​TCC	GAT​GGC​AGT​AGC​TGC​GCT​GAT​A
*MMP1*	ATG​AAG​CAG​CCC​AGA​TGT​GGA​G	TGG​TCC​ACA​TCT​GCT​CTT​GGC​A
*MMP2*	AGC​GAG​TGG​ATG​CCG​CCT​TTA​A	CAT​TCC​AGG​CAT​CTG​CGA​TGA​G
*MMP3*	CAC​TCA​CAG​ACC​TGA​CTC​GGT​T	AAG​CAG​GAT​CAC​AGT​TGG​CTG​G
*MMP9*	TTC​CAA​ACC​TTT​GAG​GGC​GA	CAA​AGG​CGT​CGT​CAA​TCA​CC
*MMP14*	CCT​TGG​ACT​GTC​AGG​AAT​GAG​G	TTC​TCC​GTG​TCC​ATC​CAC​TGG​T
*FGF2*	GAA​GAG​TGA​GCC​CCT​GAT​TG	CCT​TTG​ATA​GAC​ACA​ACT​CCT​CTC
*angiopotietin-1*	CAA​CAG​TGT​CCT​TCA​GAA​GCA​GC	CCA​GCT​TGA​TAT​ACA​TCT​GCA​CAG
*GAPDH*	CCA​CAG​TCC​ATG​CCA​TCA​CT	GGC​AGG​GAT​GAT​GTT​CTG​GAG

### 2.5 Western blotting analysis

Western blotting (WB) analysis was conducted as previously reported (Nishinaka et al., 2023). Briefly, the cells were rinsed with ice-cold phosphate-buffered saline (PBS) and lysed using radioimmunoprecipitation assay buffer supplemented with protease inhibitors (2 μg/mL antipain, leupeptin, and aprotinin) obtained from Peptide Institute Inc. (Osaka, Japan) and Nacalai Tesque. Protein concentrations were measured using the Bradford assay (Bio-Rad Laboratories). The lysates were combined with 6X reducing sample buffer, heated to 95 °C for 5 min, and subjected to sodium dodecyl sulfate-polyacrylamide gel electrophoresis. Subsequently, proteins were transferred onto polyvinylidene difluoride membranes (Merck Millipore, Burlington, MA, United States). Membranes were blocked in 5% skim milk prepared in Tris-buffered saline-0.05% Tween 20 for 1 h at room temperature and then incubated with primary antibodies overnight at 4°C. Following three washes with Tris-buffered saline-0.05% Tween 20, membranes were treated with secondary antibodies for 1 h at room temperature. Chemiluminescence signals were detected using Amersham ECL Prime reagents (Clytia, Marlborough, MA, United States) and visualized with the Amersham Imager 600 (Clytia). Band intensities were quantified using ImageJ software (National Institutes of Health, Bethesda, MD, United States).

### 2.6 Immunostaining assay

To prepare EA. hy926 cells for imaging, they were fixed in 4% paraformaldehyde for 20 min, permeabilized with 0.1% Triton X-100 in PBS for 20 min, and blocked with 3% BSA in PBS for 1 h. After blocking, the cells were incubated overnight at 4 °C with a polyclonal goat anti-integrin αVβ8 antibody (1:500 dilution). Subsequently, an Alexa Fluor 488-conjugated secondary antibody (1:1,000 dilution; Thermo Fisher Scientific) was applied for 1 h at room temperature, and the cells were counterstained with DAPI (1.43 μM) and phalloidin for 10 min each. Images were obtained with a BZ-X710 microscope (Keyence, Osaka, Japan) at ×60 magnification using immersion oil.

### 2.7 Tube formation assay

In this study, the tube formation assay protocol was modified based on previously published methodologies to assess angiogenic capacity *in vitro* (Hatipoglu et al., 2023). In this protocol, growth factor-reduced Matrigel (Corning, Corning, New York, United States) was used to coat μ-Slide Angiogenesis chambers (ibidi GmbH, Gräfelfing, Germany) with 10 μL of Matrigel to create a uniform, flat surface for cell seeding. The gel was solidified by incubation at 37°C for 30 min. EA. hy926 cells were cultured in a medium containing 10% fetal bovine serum (FBS) until 70%–80% confluence was achieved. Before experimentation, the medium was replaced with a growth medium containing 3% FBS for 24 h. Cells were then trypsinized using Trypsin EDTA (Gibco), centrifuged at 500 × *g* for 5 min, and resuspended in a medium supplemented with 3% FBS. For the assay, cells were pretreated with 100 ng/mL TNF-α for 6 h with or without 75 μg/mL HRG. A total of 5 × 10^3^ cells were plated per well in a final volume of 50 μL. The slides were incubated at 37 °C under 5% CO_2_ and 95% humidity for 16 h. Following incubation, cells were stained with 8 μg/mL calcein acetoxymethyl ester (Corning, 354,217). Images were captured using a BZ-X710 microscope (Keyence) at ×4 magnification, and total tube lengths were quantified using the Angiogenesis Analyzer tool available in ImageJ/FIJI software.

### 2.8 Transfection of EA.hy926 cells with siRNAs

EA.hy926 cells were plated for transfection experiments and cultured until they achieved 70%–80% confluence. Transfection was conducted using 50 nM of siRNA targeting integrin αV or β8, or a control siRNA, with Lipofectamine RNAiMAX Reagent (Invitrogen, Carlsbad, CA, United States) following the manufacturer’s instructions. Post-transfection, the cells were maintained for 24 h prior to further treatments. Subsequently, cells were exposed to 100 ng/mL TNF-α for 4.5 h, with or without the treatment, to simulate inflammatory responses.

### 2.9 Flow cytometry analysis

Flow cytometry analysis was performed using a previously established protocol with slight modifications (Nishinaka et al., 2022). Briefly, EA. hy926 cells were seeded at a density of 5.0 × 10^4^ cells per 500 μL in 24-well plates and treated with TNF-α, HRG, and an NRF2 activator at specified concentrations for predetermined time intervals. Post-treatment, cells were collected by washing with PBS and detaching them using Accutase Cell Detachment Solution (AT104, Innovative Cell Technologies, San Diego, CA, United States). The collected cells were rinsed twice in fluorescence-activated cell sorting (FACS) wash buffer containing PBS, 2.5% normal horse serum, 0.1% sodium azide, and 10 mM HEPES, and centrifuged at 200 × *g* for 5 min at 4 °C. Subsequently, the cells were incubated with an anti-integrin αVβ8 antibody on ice for 30 min, washed, and centrifuged again in the same wash buffer. They were then stained with a donkey anti-goat IgG Alexa Fluor 488-conjugated secondary antibody (Thermo Fisher Scientific) on ice for 20 min, washed, and centrifuged once more. The final pellet was resuspended in 100 μL PBS and stained with 2 μg/mL propidium iodide (Dojindo Laboratories) to exclude non-viable cells. Samples were analyzed using a FACSCanto II System (BD Biosciences, San Jose, CA, United States), and the data were processed with FACSDiva software (BD Biosciences) to calculate the mean fluorescence intensity of the samples.

### 2.10 Statistical analysis

Data are represented as mean ± standard deviation, with *p* < 0.05 considered significant. Group differences were analyzed using analysis of variance followed by appropriate *post hoc* tests. Each experiment was performed in triplicate or more.

## 3 Results

### 3.1 TNF-α promotes tube formation in endothelial cells in a dose-dependent manner

**FIGURE 1:**
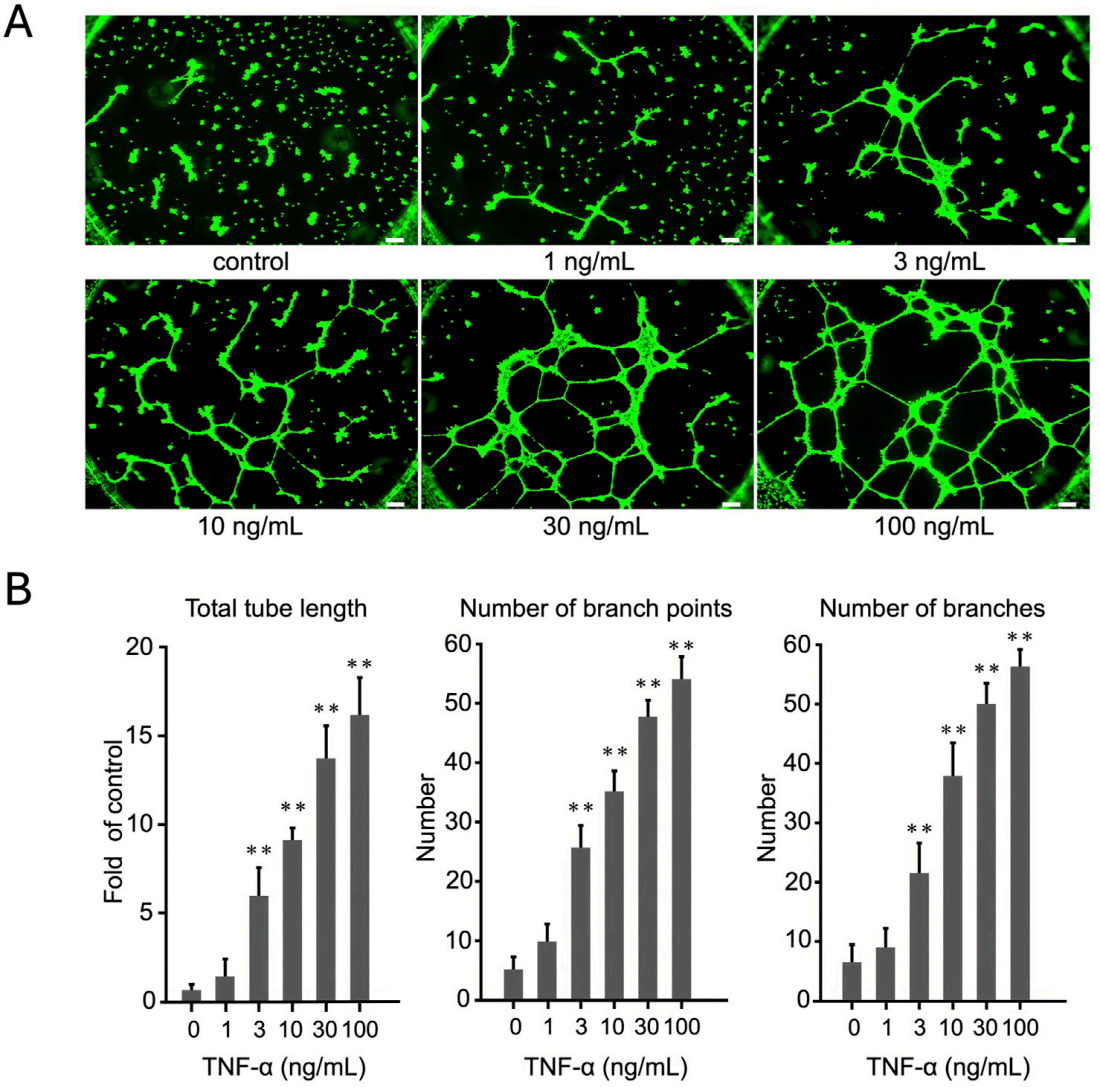
Evaluation of dose-dependent effects of TNF-α on EA.hy926 cells using tube formation assay. **(A)** Cells were treated with TNF-α at concentrations ranging from 1 to 100 ng/mL. The images display the extent of tube formation under each condition. **(B)** Number of branches, number of branch points, and total tube length were quantified and expressed as mean ± SD of values from three independent experiments (*n* = 3). Statistical analysis was conducted using one-way analysis of variance (ANOVA), complemented by Dunnett’s *post hoc* test, indicating notable differences between the control group and the TNF-α-treated groups (*p* < 0.01). Scale bar: 200 μm.

We used the tube formation assay to investigate the effect of different concentrations of TNF-α on tube formation in human endothelial cells, EA. hy926 and HUVECs. The cells were seeded in 24-well plates a day before the experiment. Before starting the experiment, the cells were treated with TNF-α at concentrations ranging from 1 to 100 ng/mL for 6 h. Thereafter, the cells were detached using trypsin and then seeded onto Matrigel for tube formation analysis. Figure 1 demonstrates that the total tube length of EA. hy926 cells increased proportionally with increasing concentrations of TNF-α. Similarly, HUVECs exhibited a dose-dependent rise in total tube length as TNF-α concentration increased (Supplementary Figure S1). These observations demonstrate that TNF-α stimulates tube formation in human endothelial cells, with the effect increasing proportionally to its concentration. As the highest effect was observed at 100 ng/mL, all our experiments were performed using this TNF-α concentration. In addition, because of the ease of transfection, similarity of results to that obtained for HUVECs, and common use in many studies, only EA. hy926 cells were used in the subsequent experiments (Yu et al., 2019). We also examined how cell numbers affected tube formation. As the number of cells increased, tube formation occurred even without stimulation. Therefore, in other experiments, we used 5,000 cells per well when stimulated with TNF alone (Supplementary Figure S2).

### 3.2 TNF-α induces integrin αV and β8 expression in EA.hy926 cells

**FIGURE 2:**
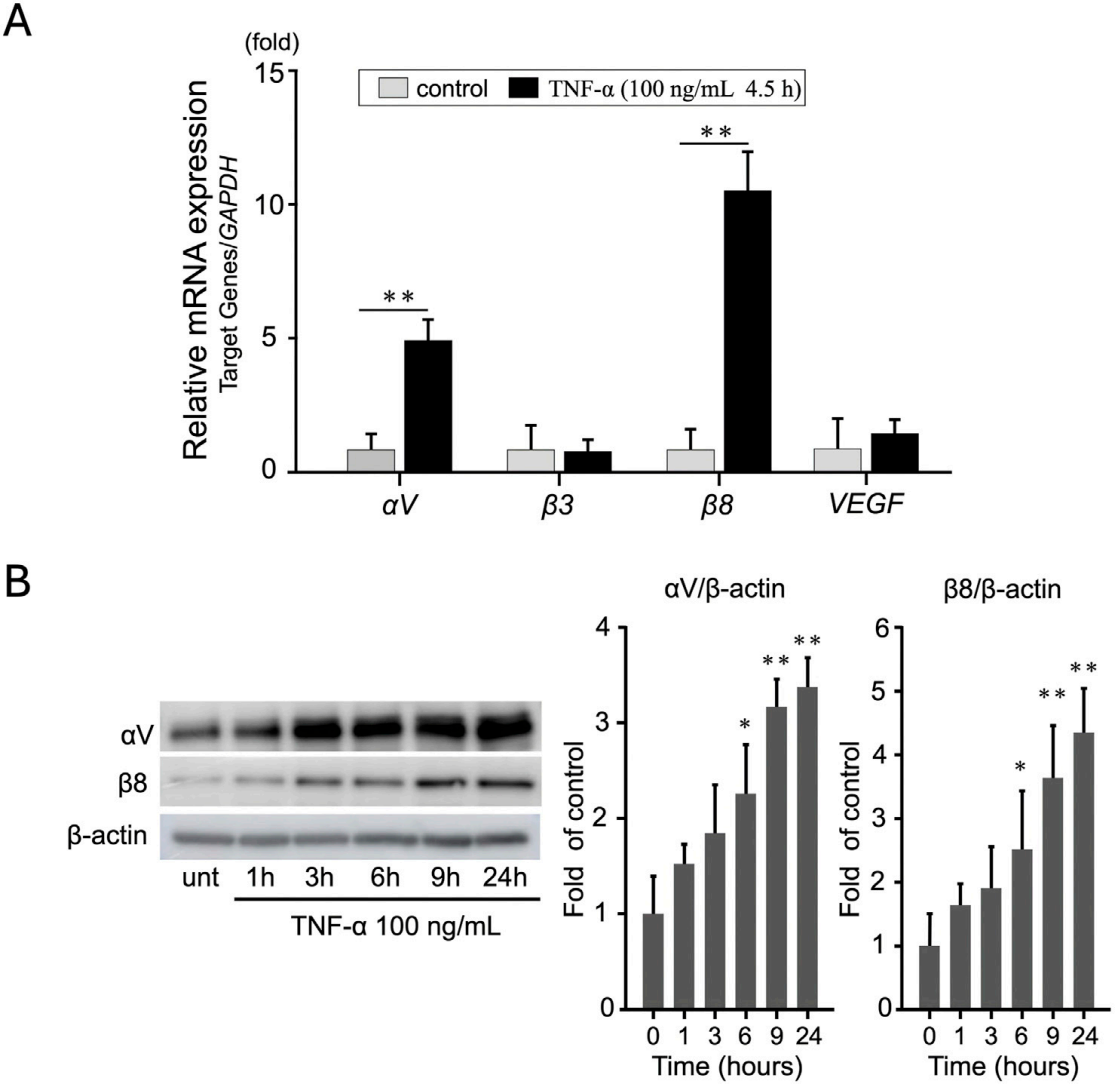
TNF-α induces integrin αV and β8 expression in EA. hy926 cells. **(A)** Relative mRNA expression of integrins αV, β3, and β8 in EA. hy926 cells treated with 100 ng/mL TNF-α for 4.5 h. mRNA levels were measured using qRT-PCR and normalized against *GAPDH* levels. The results are expressed as mean ± SD based on data from three independent experiments (*n* = 3). **p < 0.01 when compared to the control group. To assess the differences among the groups, an independent samples t-test was employed for statistical analysis. **(B)** Western blots show an increase in integrin αV and β8 protein levels. Data are presented as mean ± SD of values from three independent experiments (*n* = 3). To determine statistical significance, one-way ANOVA was performed, followed by Tukey’s *post hoc* analysis.*p < 0.05, **p < 0.01 compared to the control.

To determine the effect of TNF-α on the expression of proangiogenic genes in EA.hy926 cells, we treated the cells with 100 ng/mL TNF-α. The expression levels were measured using qRT-PCR and normalized against GAPDH expression. Among the selected genes, integrin αV and β8, but not integrin β3 and VEGF, showed the most significant increase at 4.5 h (Figure 2A). In particular, the expression of integrin αV increased approximately 5-fold (*p* < 0.01) and that of integrin β8 increased approximately 10-fold (*p* < 0.01) following TNF-α treatment. These results were further confirmed using WB analysis (Figure 2B). In addition to these integrins, we also evaluated the expression of other angiogenesis-related genes but did not find any significant change (Supplementary Figure S3). These findings indicate that TNF-α selectively upregulates the expression of integrin αV and β8 in EA. hy926 cells.

### 3.3 RNAi suppression of integrin αV and β8 reduces TNF-α–induced tube formation

**FIGURE 3:**
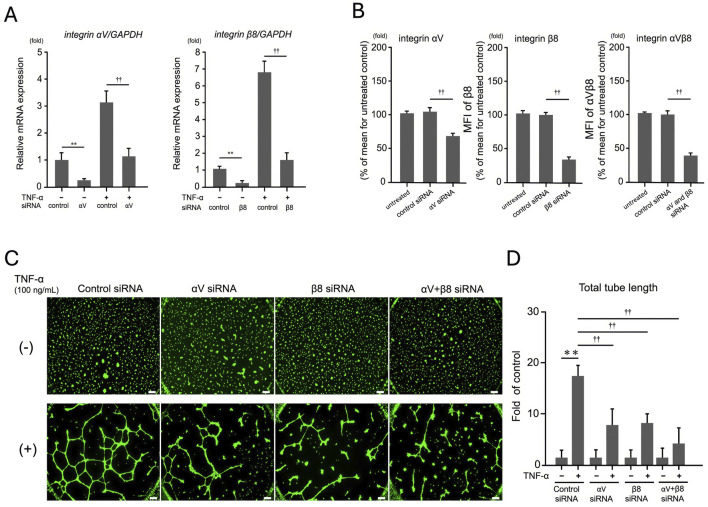
Effect of integrin αV and β8 on TNF-α-dependent tube formation in EA. hy926 cells. Cells were transfected with siRNAs targeting integrins αV and β8. **(A)** qRT-PCR analysis of the extent of suppression of target genes integrin αV and β8 by RNAi. After 24 h, αV siRNA suppressed the expression of the αV gene by 81% and β8 siRNA suppressed the expression of the β8 gene by 94%. Tukey’s *post hoc* test was used for statistical analysis (**p < 0.01, ††*p* < 0.01), and the data are presented as mean ± SD from three independent experiments (*n* = 3). **(B)** FACS analysis of integrin αV and β8 protein expression. Mean fluorescence intensity (MFI) of integrin αV, β8, and αVβ8 was measured in EA. hy926 cells transfected with control siRNA, αV siRNA, β8 siRNA, or both. Knockdown of αV and β8 significantly reduced the protein levels of these integrins (p < 0.01), further confirming the efficiency of RNAi-mediated suppression. **(C)** Tube formation assays involved EA. hy926 cells treated with control, control siRNA, αV siRNA, β8 siRNA, or αV + β8 siRNA, followed by TNF-α (100 ng/mL) stimulation. Scale bar: 200 μm. **(D)** TNF-α treatment significantly enhanced the tube length compared with that in the control siRNA treatment, but this increase was notably mitigated by treatment with αV and β8 siRNAs. Results are expressed as mean ± SD from three independent experiments (*n* = 3). (**p < 0.01, ††*p* < 0.01; Tukey’s *post hoc* test).

To evaluate the role of integrin αV and β8 on TNF-α–dependent tube formation in EA.hy926 cells, the cells were transfected with siRNA targeting integrin αV and β8 using RNAiMAX. First, qRT-PCR was performed to determine the extent of RNAi-mediated suppression of the target genes integrin αV and β8. Control siRNA did not affect the expression of these genes (data not shown). After 24 h, αV siRNA suppressed the expression of the αV gene by 81% and that of the β8 gene by 94% compared to control siRNA, indicating that this method was effective in inhibiting the expression of both the integrin genes (Figure 3A) without affecting cell viability (Supplementary Figure S4A). Additionally, FACS analysis confirmed that RNAi-mediated knockdown significantly reduced the protein levels of integrin αV and β8 (p < 0.01, Figure 3B), further validating the effectiveness of siRNA-mediated suppression at the protein level.

To further investigate the role of these integrins in TNF-α–induced angiogenesis, we performed the tube formation assay. As shown in Figures 3C, D, knockdown of integrin αV and β8 significantly reduced TNF-α–induced tube formation, as evidenced by decreased total tube length compared with that in the control. These results indicate that targeting integrin αV and β8 can effectively reduce TNF-α–induced tube formation in endothelial cells, highlighting the potential role of these integrins in TNF-α–mediated angiogenic processes.

### 3.4 HRG reduces TNF-α-stimulated tube formation and integrin expression in EA.hy926 cells

**FIGURE 4:**
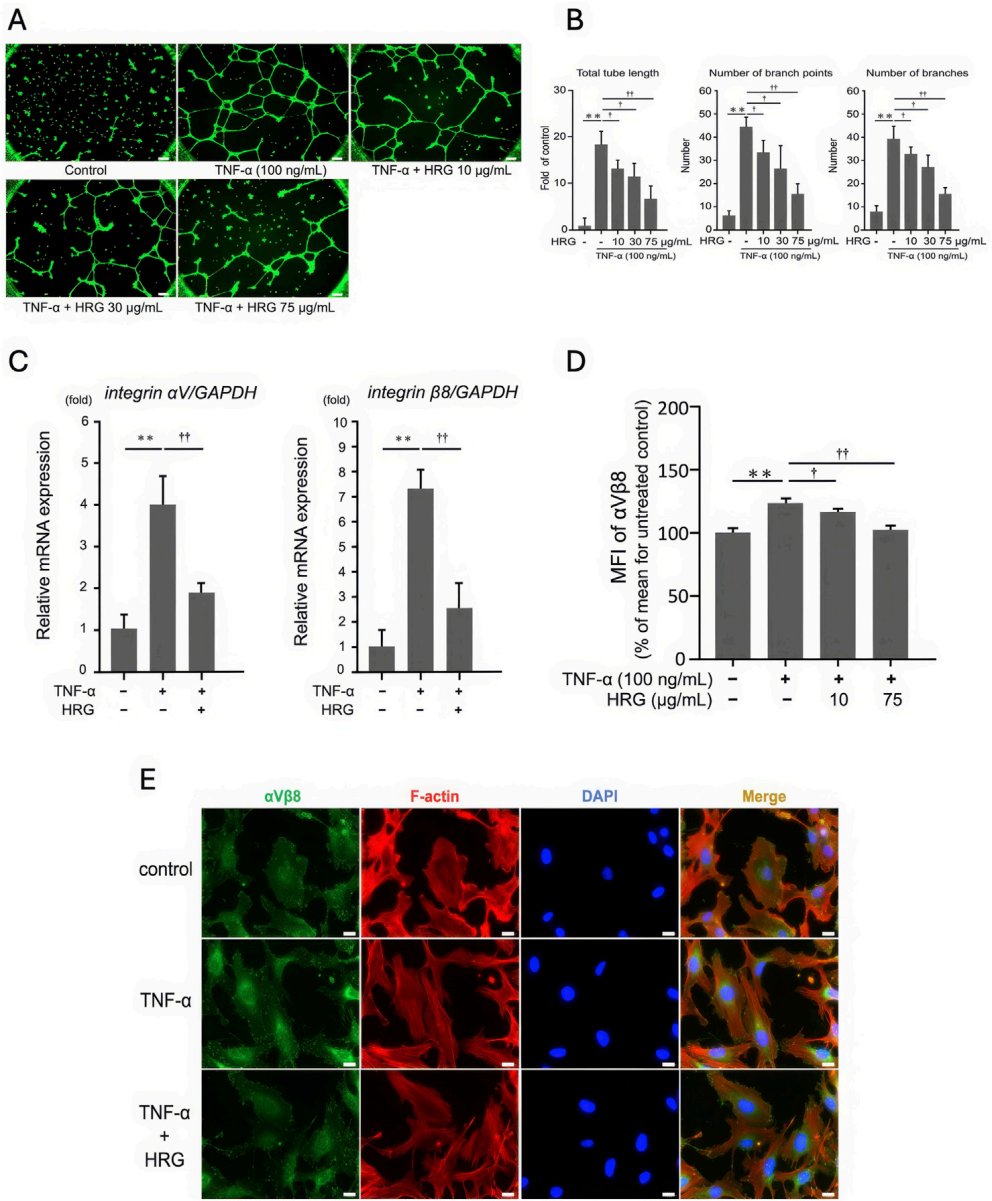
The effect of HRG on TNF-α-induced integrin expression and tube formation in EA. hy926 cells. **(A)** Representative images of tube formation in EA. hy926 cells treated with TNF-α (100 ng/mL) and various concentrations of HRG (10, 30, and 75 μg/mL). Scale bar: 200 μm. **(B)** Treatment with HRG at 10, 30, and 75 μg/mL progressively reduced TNF-α-induced tube formation, as evidenced by a decrease in number of branches, number of branch points, and total tube length. Data are shown as mean ± SD from three independent experiments (*n* = 3). (**p < 0.01, †*p* < 0.05, ††*p* < 0.01; Tukey’s *post hoc* test). **(C)** qRT-PCR analysis showing the relative mRNA levels of integrin αV and β8 in EA. hy926 cells treated with TNF-α and HRG. TNF-α treatment significantly upregulated the mRNA levels of integrin αV and β8, whereas cotreatment with HRG markedly suppressed this upregulation (**p < 0.01, ††*p* < 0.01; Tukey’s *post hoc* test). **(D)** FACS analysis of the cell surface expression of integrin αVβ8 in EA.hy926 cells treated with TNF-α and HRG. The mean fluorescence intensity (MFI) values indicate that TNF-α significantly increased the expression of integrin αVβ8, which was significantly reduced by cotreatment with HRG. Data represent mean ± SD from three independent experiments (*n* = 3) (**p < 0.01, †*p* < 0.05, ††*p* < 0.01; Tukey’s *post hoc* test). **(E)** Immunofluorescence images illustrating the impact of HRG on TNF-α-induced integrin αVβ8 expression in EA. hy926 cells. Cells were treated with TNF-α (100 ng/mL) with or without HRG. Immunostaining was performed for integrin αVβ8 (green), F-actin using phalloidin (red), and nuclei with DAPI (blue). Merged images show the colocalization of these markers. HRG treatment reduced the TNF-α–induced increase in the expression of integrin αVβ8. Scale bars: 200 μm.

To evaluate the role of HRG on TNF-α–induced expression of integrins and tube formation in EA.hy926 cells, the cells were pretreated with different concentrations of HRG for 2 h, followed by treatment with 100 ng/mL TNF-α for 6 h. HRG cotreatment markedly inhibited TNF-α-induced tube formation, as reflected by the significant decrease in total tube length, the number of branch points, and the overall branch count (Figures 4A, B), without affecting cell viability (Supplementary Figures S4B, C). At the molecular level, HRG cotreatment significantly inhibited TNF-α–induced mRNA expression of integrin αV and β8 (Figure 4C). FACS analysis further confirmed this finding wherein a significant decrease in the mean fluorescence intensity of integrin αVβ8 on the cell surface was noted, indicating that HRG not only impacts mRNA expression but also reduces the protein levels of these integrins (Figure 4D). This suppression was also visually confirmed using immunofluorescence staining for integrin αVβ8 (green), F-actin (phalloidin, red), and nuclei (DAPI, blue) (Figure 4E). Together, these results indicate that HRG significantly inhibits TNF-α–induced integrin αV and β8 mRNA and protein expression, as well as tube formation, underscoring the potential of HRG as a potent antiangiogenic agent via the modulation of integrin-mediated signaling pathways.

### 3.5 HRG-induced NRF2 translocation and suppression of TNF-α–induced integrin expression and tube formation

**FIGURE 5:**
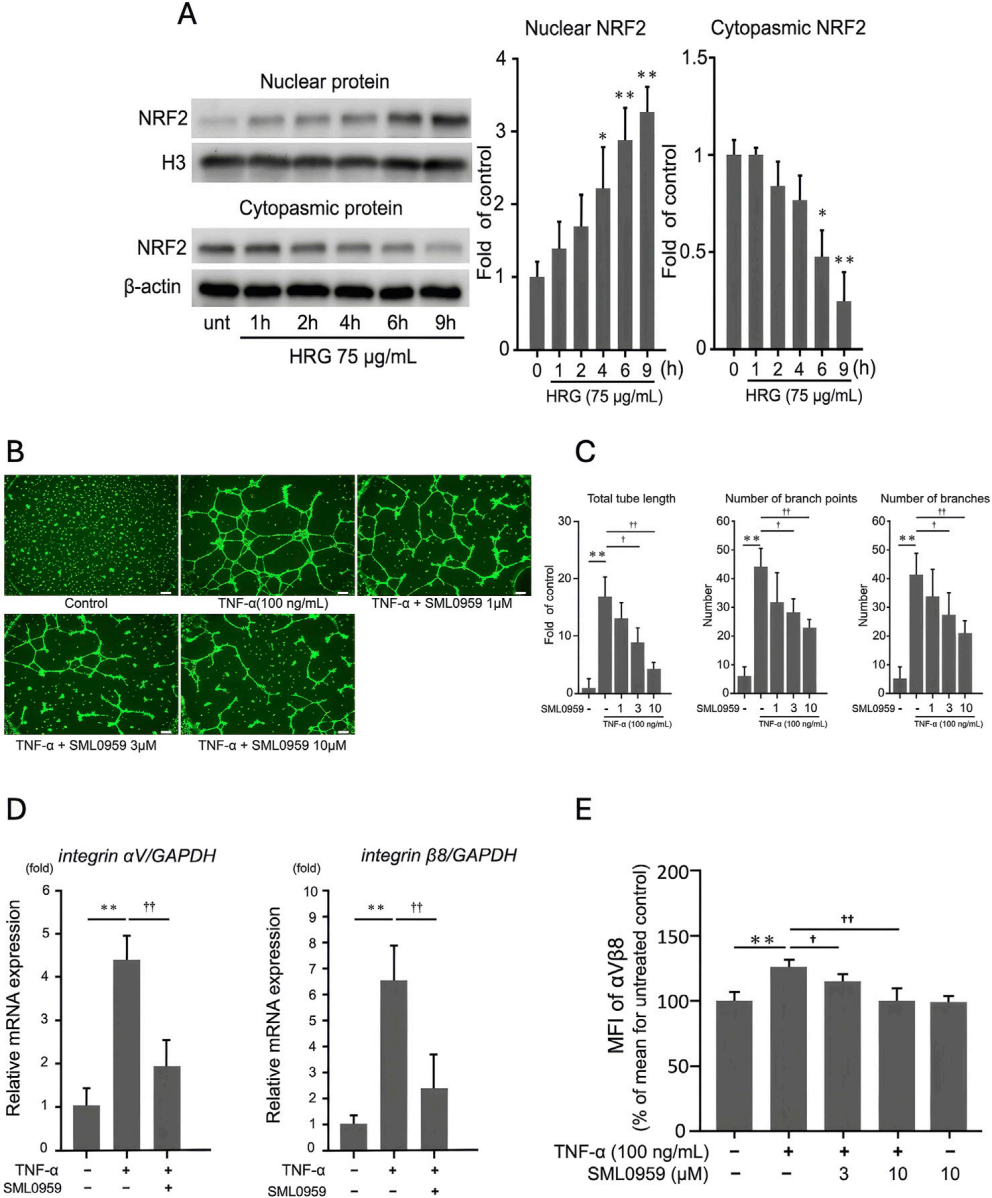
HRG-induced nuclear translocation of NRF2 and its inhibitory effects on TNF-α-induced angiogenic processes in EA.hy926 cells. **(A)** Analysis of NRF2 expression via Western blotting in the nuclear and cytoplasmic fractions of EA. hy926 cells treated with HRG (75 μg/mL) over different time points (1, 2, 4, 6, and 9 h). Loading controls for nuclear and cytoplasmic proteins were histone H3 and β-actin, respectively. HRG treatment induced translocation of NRF2 to the nucleus in a time-dependent manner; unt: untreated. **(B)** Representative images of tube formation in EA. hy926 cells treated with TNF-α (100 ng/mL) and various concentrations of SML0959, an NRF2 activator (1, 3, and 10 μM). Scale bar: 200 μm. **(C)** Number of branches, number of branch points, and total tube length under each condition. Treatment with NRF2 activator significantly reduced TNF-α–induced tube formation in a dose-dependent manner. Data are presented as mean ± SD of values from three independent experiments (*n* = 3) (**p < 0.01, †*p* < 0.05, ††*p* < 0.01; Tukey’s *post hoc* test). **(D)** qRT-PCR analysis showing the relative mRNA levels of integrin αV and β8 in EA. hy926 cells treated with TNF-α and NRF2 activator. Treatment with an NRF2 activator significantly reduced the TNF-α–induced mRNA levels of integrin αV and β8. Data are presented as mean ± SD of values from three independent experiments (*n* = 3) (**p < 0.01, ††*p* < 0.01; Tukey’s *post hoc* test). **(E)** FACS analysis of the cell surface expression of integrin αVβ8 in EA. hy926 cells treated with TNF-α and NRF2 activator. The MFI values indicate that TNF-α significantly increased the expression of integrin αVβ8, which was significantly reduced by cotreatment with NRF2 activator. Data are presented as mean ± SD of values from three independent experiments (*n* = 3). (**p < 0.01, †*p* < 0.05, ††*p* < 0.01; Tukey’s *post hoc* test).

We hypothesized that HRG reduces TNF-α–induced integrin expression and tube formation by activating NRF2, a critical regulator of oxidative stress and inflammation. To investigate this, EA. hy926 cells were treated with 75 μg/mL HRG for various durations (1, 2, 4, 6, and 9 h), and the NRF2 levels were analyzed in cytoplasmic and nuclear fractions using WB (Figure 5A). HRG induced a time-dependent translocation of NRF2 to the nucleus, with peak levels observed at 9 h. To further examine whether NRF2 activation inhibits TNF-α-induced angiogenic responses, we cotreated endothelial cells with TNF-α and the NRF2 activator SML0959 at concentrations that did not affect cell viability (Supplementary Figure S4D). This treatment significantly suppressed TNF-α-induced tube formation, as evidenced by a reduction in total tube length, branch points, and branch numbers (Figures 5B, C). At the molecular level, NRF2 activation significantly inhibited TNF-α-induced upregulation of integrin αV and β8 mRNA (Figure 5D). This effect was further confirmed using FACS analysis wherein a decrease in the mean fluorescence intensity of integrin αVβ8 on the cell surface was evident (Figure 5E). Together, these findings indicate that HRG activates NRF2, and this activation effectively suppresses TNF-α–induced integrin αV and β8 expression, as well as tube formation.

Thus, the activation of the NRF2 pathway by HRG presents a novel mechanism through which HRG exerts its antiangiogenic effects, highlighting the potential of HRG as a therapeutic agent targeting integrin-mediated signaling in TNF-α-driven angiogenesis.

## 4 Discussion

In this study, we explored the antiangiogenic effects of HRG on TNF-α-stimulated human endothelial cells through the tube formation assay, a commonly employed *in vitro* model. This assay is widely recognized for its ability to replicate *in vivo* angiogenesis by facilitating capillary-like structure formation in endothelial cells (Arnaoutova et al., 2009; Arnaoutova et al., 2010). The tube formation assay is critical for understanding the angiogenic process, as it provides both a functional readout and insights into the complex signaling pathways involved.

We demonstrated that TNF-α promotes tube formation in EA.hy926 endothelial cells in a dose-dependent manner (Figure 1), highlighting its crucial role as a proinflammatory cytokine driving pathological angiogenesis. We further confirmed that the influence of TNF-α on tube formation is mediated by the increased expression of integrins αV and β8, as shown in qRT-PCR and WB analyses (Figure 2). RNAi-mediated targeting of these integrins significantly reduced TNF-α–induced tube formation (Figure 3C), indicating that integrins αV and β8 play a central role in orchestrating the responses of endothelial cells to TNF-α. These findings indicate that inhibiting integrins αV and β8 using RNAi technology suppresses TNF-α–induced endothelial cell responses, underscoring their potential as therapeutic targets for controlling angiogenesis. This provides new insights into integrin-mediated mechanisms of angiogenesis and highlights the role of HRG in modulating these pathways—an area not extensively explored before.

Consistent with our findings on the critical role of integrins αV and β8 in mediating TNF-α–induced tube formation, recent studies have demonstrated the therapeutic potential of targeting αVβ8 integrin in various diseases. αVβ8 integrin plays a pivotal role in regulating TGF-β signaling, which is essential for both angiogenesis and fibrosis (Pang et al., 2023; Shihan et al., 2021). In cancer settings, αVβ8-expressing tumor cells have been shown to evade immune responses by modulating TGF-β activation in immune cells, positioning it as a promising target in immunotherapy approaches. Furthermore, αVβ8 integrin inhibition reduces tumor growth and metastasis, while enhancing T-cell infiltration and the overall immune response in solid tumors (Takasaka et al., 2018). Recently, Reichart et al. developed a highly selective cyclic peptide targeting αVβ8, demonstrating the feasibility of using αVβ8-specific inhibitors in therapeutic settings (Reichart et al., 2019). Given these insights, inhibition of αVβ8 integrin could be a promising strategy for controlling TNF-α–induced endothelial responses, further supporting the potential of integrin-targeted therapies in angiogenesis-related diseases.

In addition to integrins, other key factors, such as matrix metalloproteinases and VEGF, are well-known regulators of angiogenesis. By promoting endothelial cell migration and proliferation, matrix metalloproteinases and VEGF play pivotal roles in initiating and supporting new blood vessel formation (Neufeld et al., 1999; Rundhaug, 2005; Lamalice et al., 2007; Esfahanian et al., 2012). However, contrary to expectations, we did not find any significant change in their expression after TNF-α treatment, which indicates that TNF-α may promote tube formation through an integrin-mediated mechanism independent of VEGF signaling (Supplementary Figure S3). Integrin αVβ8, known to mediate cell–ECM interactions, may play a critical role in facilitating TNF-α–induced tube formation by promoting cytoskeletal rearrangements and migration of endothelial cells. This VEGF-independent pathway highlights the complexity of the role of TNF-α in angiogenesis and suggests that integrin-mediated signaling may be an important compensatory mechanism when classical VEGF pathways are not activated. Further investigation of the specific integrin signaling pathways involved in TNF-α–induced angiogenesis may reveal novel therapeutic targets, particularly for conditions in which VEGF inhibitors are less effective or resistance has developed. This suggests that TNF-α may directly influence the migration of endothelial cells and their adhesion to the ECM via integrin αVβ8, bypassing the need for VEGF to drive angiogenesis.

Given the critical role of integrin αVβ8 in TNF-α–induced tube formation, it is crucial to explore agents that can modulate this pathway. We found that HRG effectively suppressed TNF-α–induced tube formation in EA.hy926 cells (Figure 4A), which is indicative of its potential as a regulator of integrin-mediated angiogenic processes. HRG also significantly inhibited TNF-α–induced mRNA and protein expression of integrins αV and β8 (Figures 4B, C). These findings demonstrate that the antiangiogenic effects of HRG are primarily mediated through the suppression of integrin αV and β8 expression at both transcriptional and protein levels. This underlines the potential of HRG as a robust antiangiogenic agent by modulating integrin-mediated signaling pathways. Our group has previously reported that HRG inhibits heparin-dependent angiogenesis induced by various growth factors, including high mobility group box 1 and basic fibroblast growth factor (Wake et al., 2009). In addition, HRG inhibits tumor angiogenesis by modulating macrophage polarization and affecting key signaling pathways that control the migration and proliferation of endothelial cells (Ringvall et al., 2011). A recent study revealed a significant link between lower HRG levels and advanced stages of lung cancer, highlighting a potential role of HRG as a biomarker of cancer progression (Winiarska et al., 2019). This study is consistent with published literature and is the first to demonstrate that HRG suppresses tube formation by downregulating the expression of integrins in TNF-α–treated EA. hy926 cells, providing a novel mechanism by which HRG exerts its antiangiogenic effects.

The ability of HRG to downregulate integrin αV and β8, thereby inhibiting TNF-α–induced tube formation, underscores its potential as an antiangiogenic agent targeting integrin-mediated pathways. However, the mechanism of action of HRG likely involves multiple signaling pathways, reflecting the complexity of angiogenesis regulation.

**FIGURE 6:**
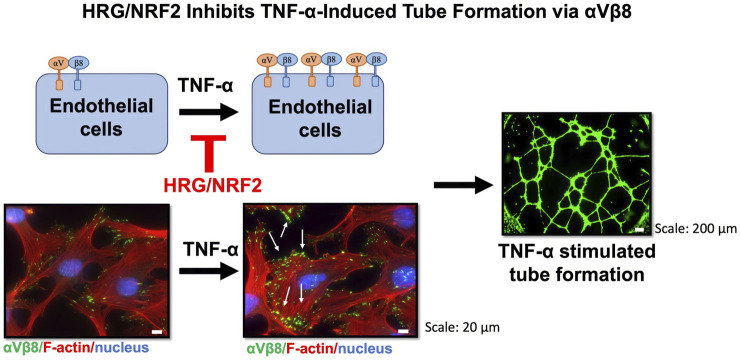
The effect of TNF-α and HRG on integrin αVβ8 expression and tube formation in EA. hy926 endothelial cells. Immunostaining was performed for integrin αVβ8 (green), actin filaments (stained with phalloidin, red), and nuclei (stained with DAPI, blue). The images show untreated cells (control, left) and cells treated with TNF-α (right). Representative images of the tube formation assay showing green fluorescence from tube-like structures formed by endothelial cells. Scale bars: 20 μm for immunostaining images and 200 μm for tube formation images. TNF-α treatment led to a significant increase in integrin αVβ8 expression and subsequent tube formation, whereas HRG/NRF2 signaling modulates this process by inhibiting integrin αVβ8 expression and angiogenesis.

Besides modulating the expression of integrins, HRG affects key molecular players in cellular stress responses and inflammation. Among these, the transcription factor NRF2 has emerged as a critical regulator of oxidative stress, with increasing evidence linking its activity to angiogenesis (Ngo and Duennwald, 2022; Hammad et al., 2023). Because angiogenesis is often associated with oxidative stress and inflammation, the interplay between HRG and NRF2 might regulate these processes. We show that HRG treatment induces nuclear translocation of NRF2 and downstream transcriptional activation (Figure 5A). To further investigate the involvement of NRF2, we used an NRF2 activator and found that its activation was associated with significant suppression of TNF-α-induced tube formation (Figures 5B, C) and expression of integrin αV and β8 (Figures 5D, E). The proposed mechanism by which HRG inhibits TNF-α-mediated tube formation through NRF2 activation is summarized in Figure 6. This figure illustrates the interplay among HRG, NRF2, and TNF-α signaling, indicating the potential pathways involved in its antiangiogenic effects. This is consistent with studies showing that the activation of the NRF2 pathway by alpinetin may exert an antiangiogenic effect by inhibiting angiogenic processes mediated by the NRF2 pathway (Zhu et al., 2021). Given the central role of NRF2 in oxidative stress response and inflammation, its activation by HRG in the context of angiogenesis opens up potential therapeutic avenues beyond cancer, including chronic inflammatory diseases in which pathological angiogenesis plays a key role.

Although our study provides compelling evidence of the antiangiogenic effects of HRG *in vitro*, further research is necessary to validate these *in vivo* findings of pathological angiogenesis. Future studies should aim at elucidating the detailed mechanisms by which integrin αVβ8 contributes to angiogenesis and determining whether HRG exerts its effects through receptor-mediated pathways or by directly binding to TNF-α. Additionally, exploring the potential receptor-mediated effects of HRG on integrin signaling could help further clarify the mechanisms underlying its antiangiogenic activity and establish it as a target for antiangiogenic therapies in various pathological conditions.

## Data Availability

The original contributions presented in the study are included in the article/Supplementary Material, further inquiries can be directed to the corresponding author.

## Abbreviations

ECM, Extracellular matrix; FACS, fluorescence-activated cell sorting; HRG, histidine-rich glycoprotein; HUVECs, human umbilical vein endothelial cells; EA.hy926, Immortalized Human Vascular Endothelial Cells; NRF2, nuclear factor erythroid 2-related factor 2; PBS, phosphate-buffered saline; qRT-PCR, real-time quantitative reverse transcription PCR; RNAi, RNA interference; TNF-α, tumor necrosis factor-α; VEGF, vascular endothelial growth factor; WB, Western blotting.
