# Supplementary material for: Histidine-rich glycoprotein inhibits TNF-α–induced tube formation in human vascular endothelial cells
Source: Front Pharmacol. 2025 Mar 21;16:1561628. doi: 10.3389/fphar.2025.1561628 (PMC11969118; doi:10.3389/fphar.2025.1561628)
Supplement: Supplementary file 1 [file DataSheet1.docx]

Supplementary Material

Histidine-Rich Glycoprotein Inhibits TNF-α–Induced Tube Formation in Human Vascular Endothelial Cells

Omer Faruk Hatipoglu^1^, Takashi Nishinaka^1^, Kursat Oguz Yaykasli^2^, Shuji Mori^3^, Masahiro Watanabe^3^, Takao Toyomura^3^, Masahiro Nishibori^4^, Satoshi Hirohata^5^, Hidenori Wake^1^*, Hideo Takahashi^1^

^1^ Department of Pharmacology, Kindai University Faculty of Medicine, Japan

^2^ Rheumatology and Immunology, Friedrich-Alexander-University Erlangen-Nürnberg, Erlangen, Erlangen, Germany

^3^ Department of Pharmacology, School of Pharmacy, Shujitsu University, Okayama, Japan

^4^ Department of Translational Research & Dug Development, Okayama University, Graduate School of Medicine, Dentistry and Pharmaceutical Sciences, Okayama, Japan

^5^ Department of Medical Technology, Graduate School of Health Sciences, Okayama University, Okayama, Japan

***Address for correspondence:**

Hidenori Wake, Department of Pharmacology, Kindai University Faculty of Medicine, 377-2 Ohno-Higashi, Osaka-Sayama, Osaka, 589-8511, Japan

Phone: +81-72-366-0221; e-mail: [wake-h.kindai@med.kindai.ac.jp](mailto:wake-h.kindai@med.kindai.ac.jp).

## Supplementary Figures


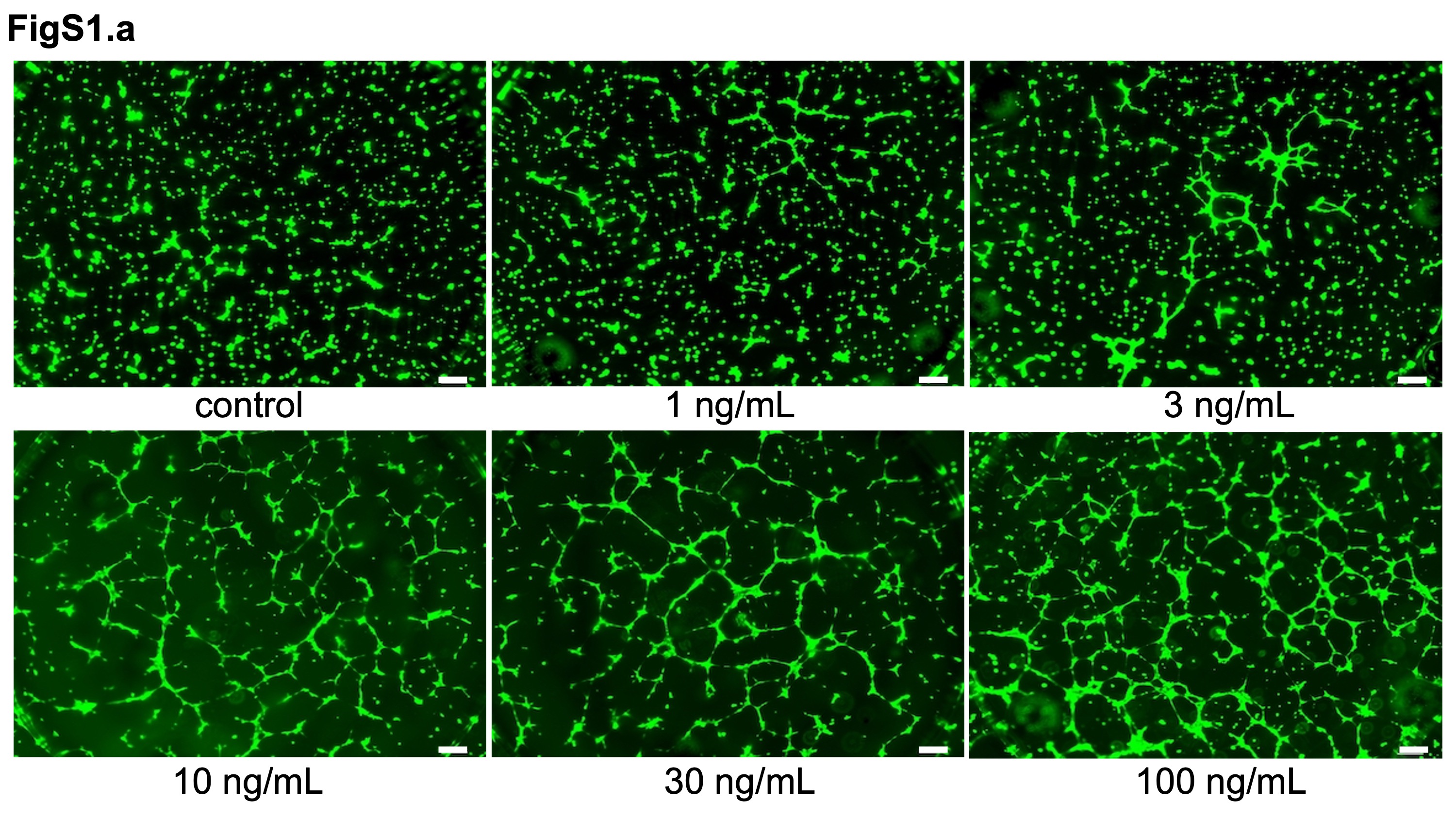


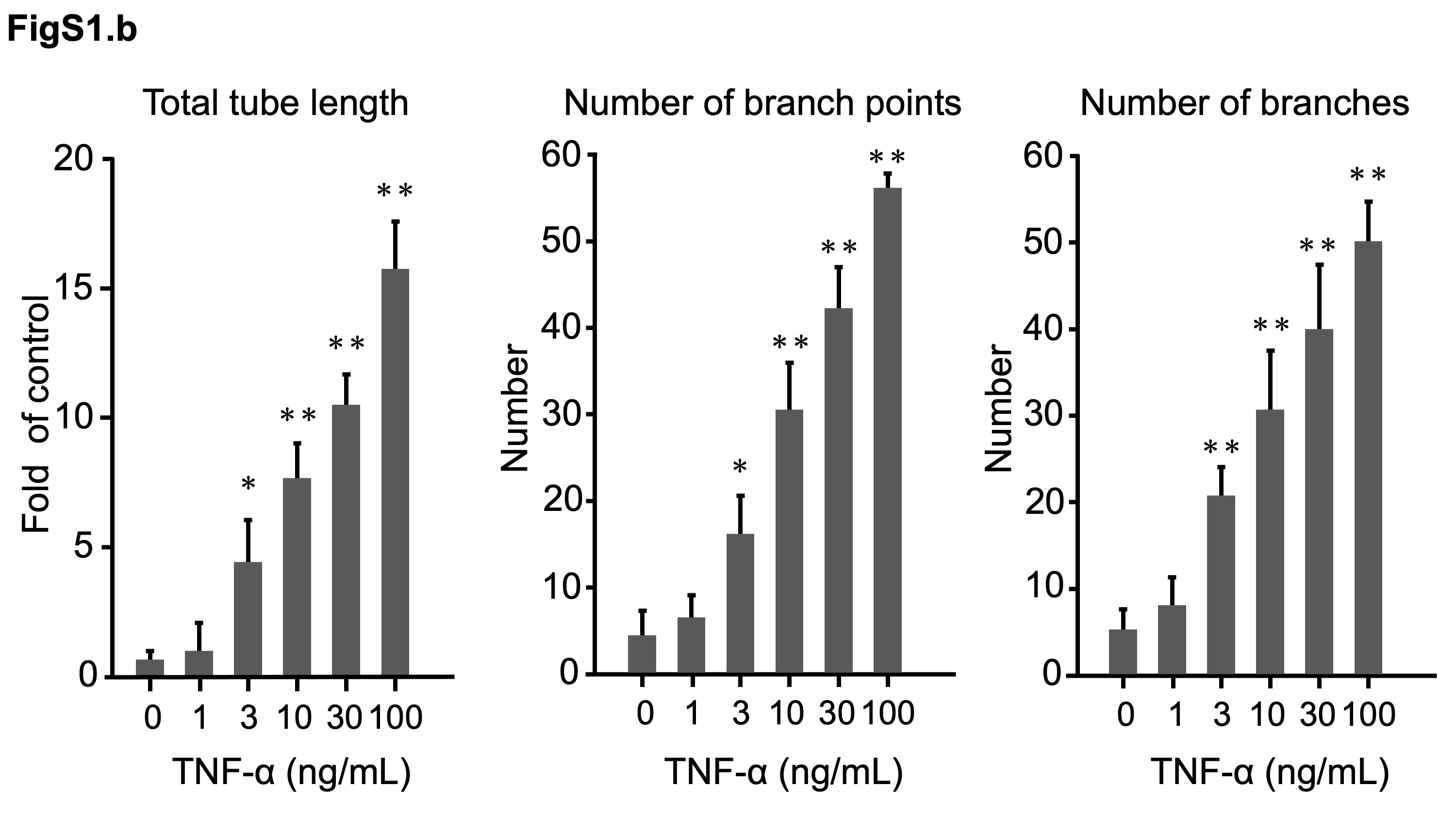


**Supplementary Figure 1. Effects of TNF-α on tube formation in HUVECs.**

**(A)** Representative images of tube formation by HUVEC cells treated with different concentrations of TNF-α (0, 1, 3, 10, 30, and 100 ng/mL). Cells were seeded on growth factor-reduced Matrigel and incubated for 6 h at 37°C in a humidified atmosphere with 5% CO_2_. Cells were stained with 8 μg/mL calcein acetoxymethylester (AM), and images were captured using a Biozero BZ-X710 microscope (Keyence, Osaka) at ×4 magnification.

**(B)** Quantification of tube formation parameters, including total tube length, number of branch points, and number of branches. The values are expressed as fold of control for total tube length and as absolute numbers for branch points and branches. Data are presented as the mean ± SD of values from three independent experiments (*n* = 3). Statistical significance was determined using one-way ANOVA followed by Tukey’s post-hoc test. Differences were considered significant at **p* **<** 0.05 and highly significant at ***p* **<** 0.01. Scale bar: 200 μm


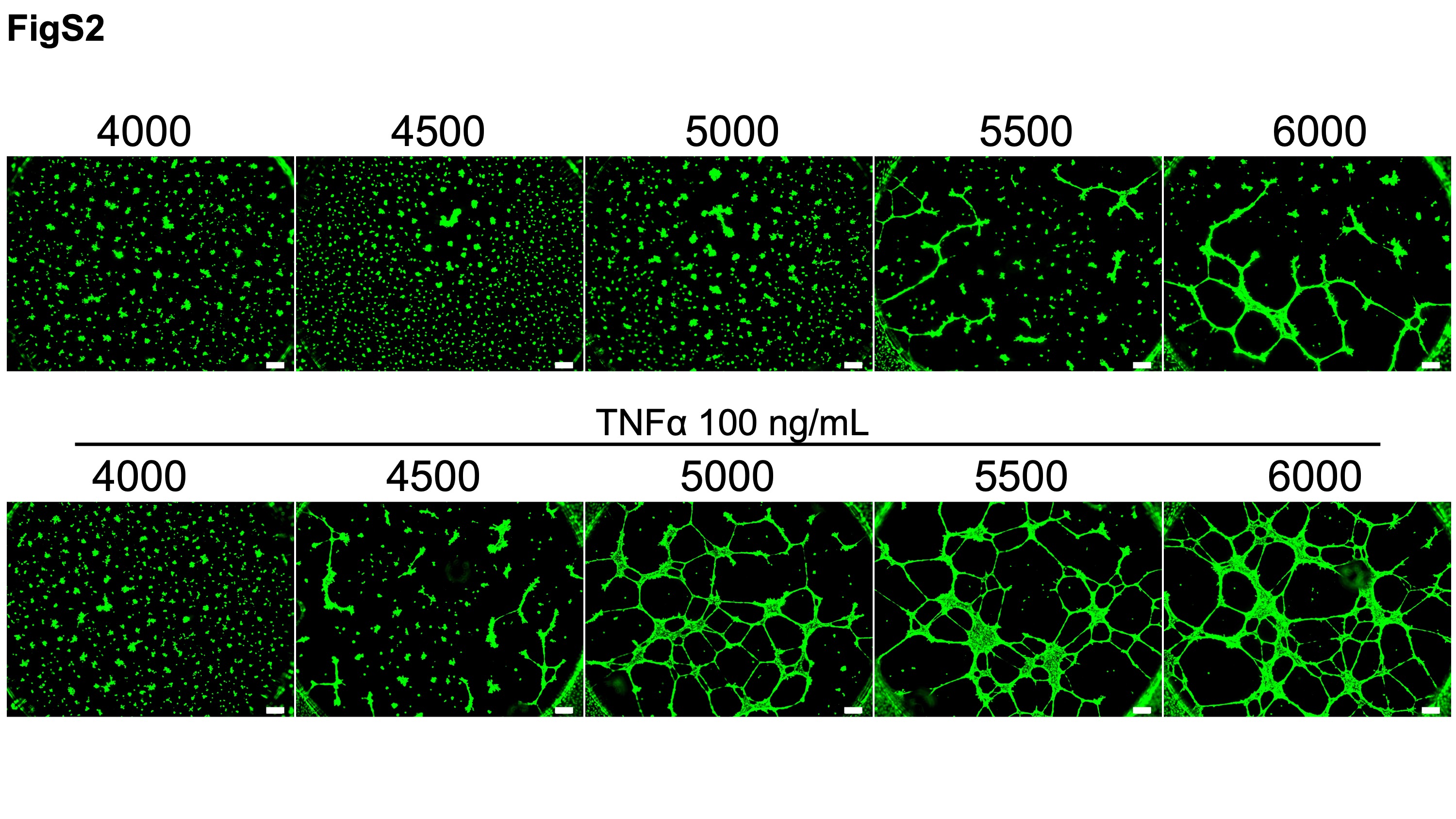


**Supplementary Figure 2. Effect of cell number on tube formation in EA.hy926 cells.**

Tube formation assays were conducted using different cell densities (4000, 4500, 5000, 5500, and 6000 cells per well) in the presence and absence of TNF-α (100 ng/mL). Top panel: Tube formation in the absence of TNF-α, showing that increased cell number promotes tube formation even without stimulation. Bottom panel: Tube formation in the presence of TNF-α, demonstrating enhanced tube formation with higher cell densities. All experiments were conducted using 3% fetal bovine serum. These results indicate that cell number significantly influences tube formation; a density of 5000 cells per well was used in subsequent experiments when stimulating with TNF-α alone. Scale bar: 200 μm


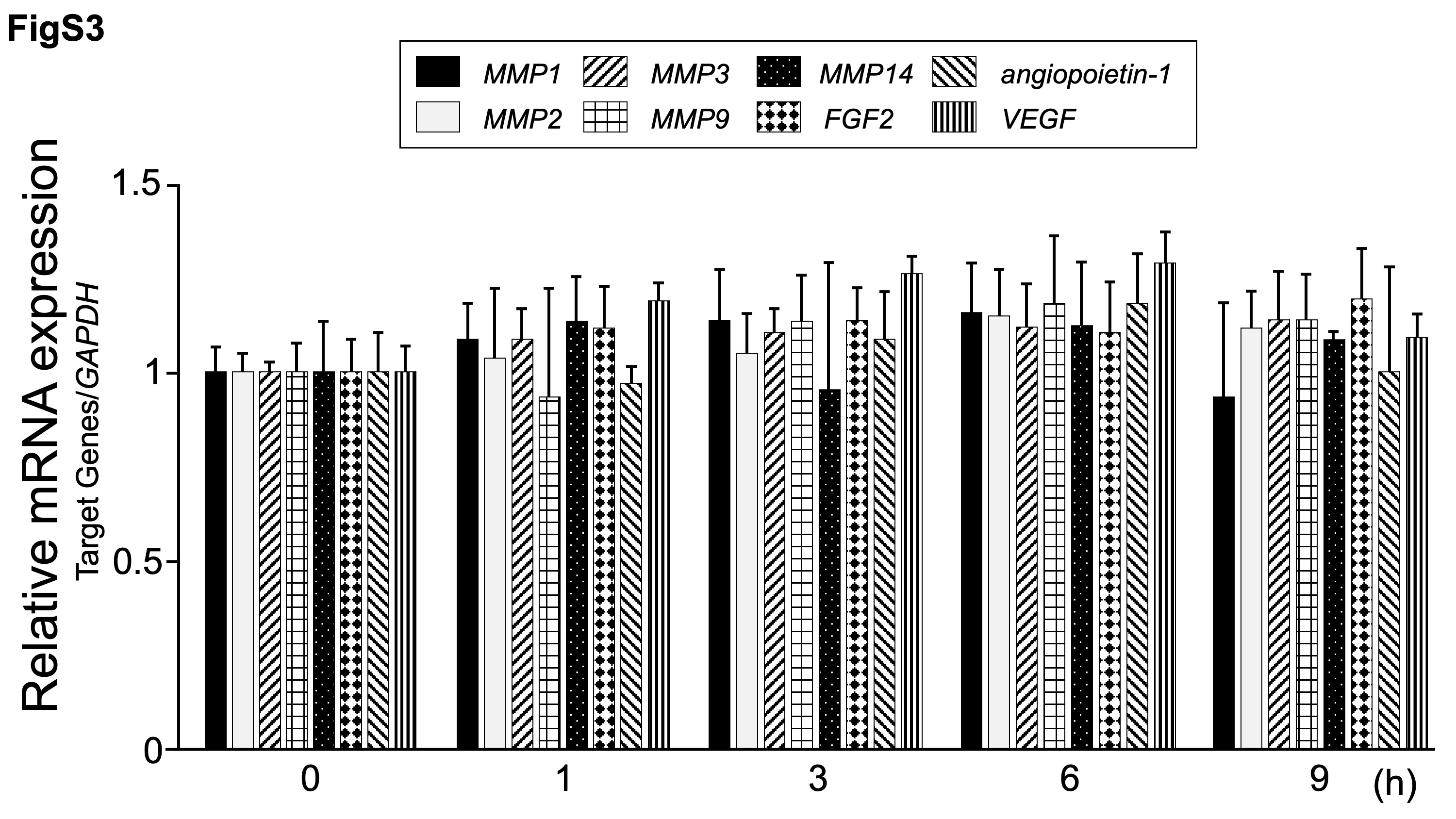


**Supplementary Figure 3. Expression levels of angiogenesis-related genes in EA.hy926 cells treated with TNF-α over time.**

EA.hy926 cells were treated with TNF-α for various time points (0, 1, 3, 6, and 9 h). The fold increase in the expression levels of MMP1, MMP2, MMP3, MMP9, MMP14, FGF2, VEGF, and angiopoietin-1 was assessed using quantitative PCR. No significant changes were observed in the expression levels of these genes across the time points. Data are presented as mean ± SD of values from three independent experiments (n = 3). Statistical analysis using one-way ANOVA followed by Tukey’s post-hoc test showed no significant differences between the groups

**
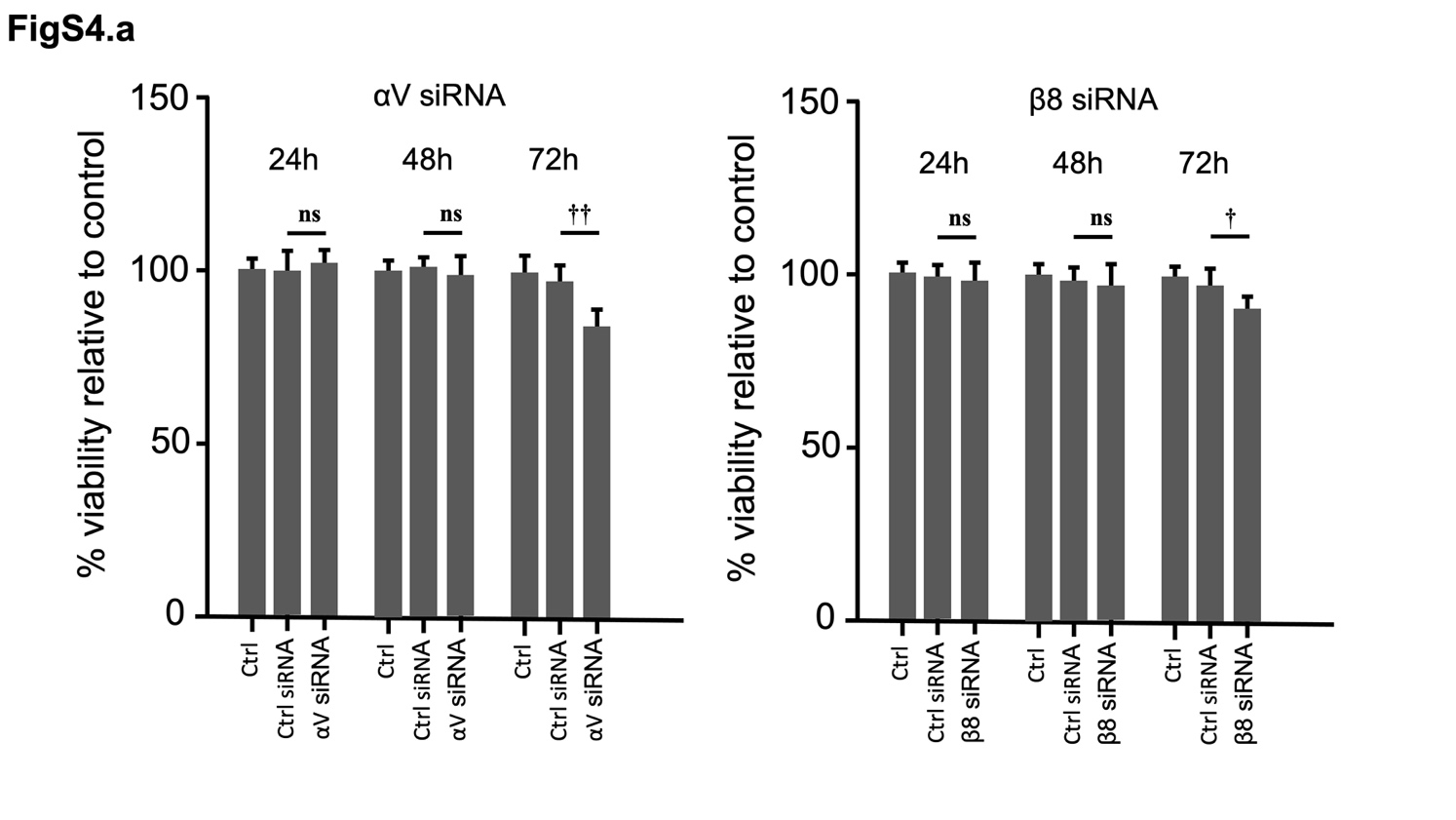

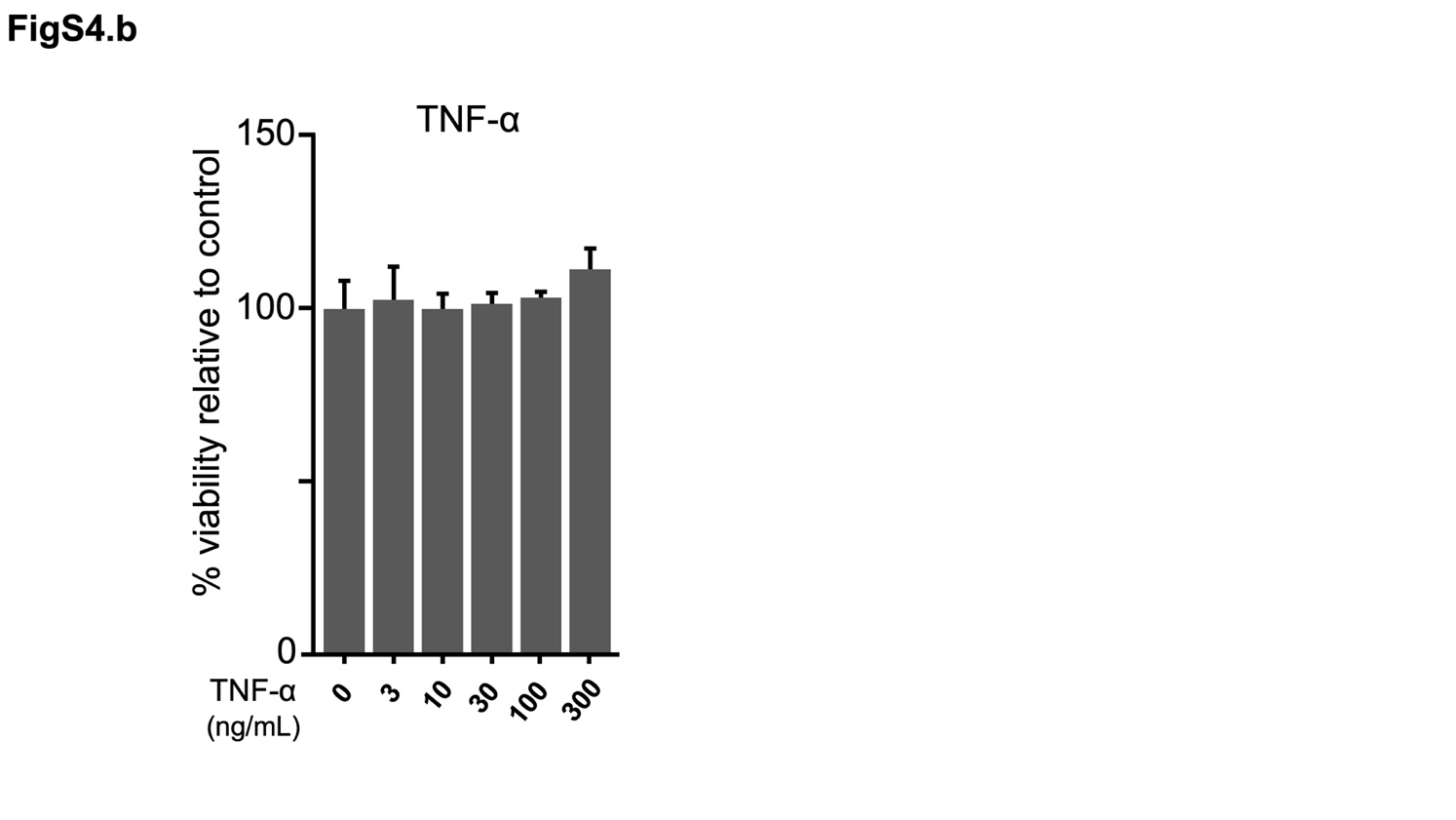

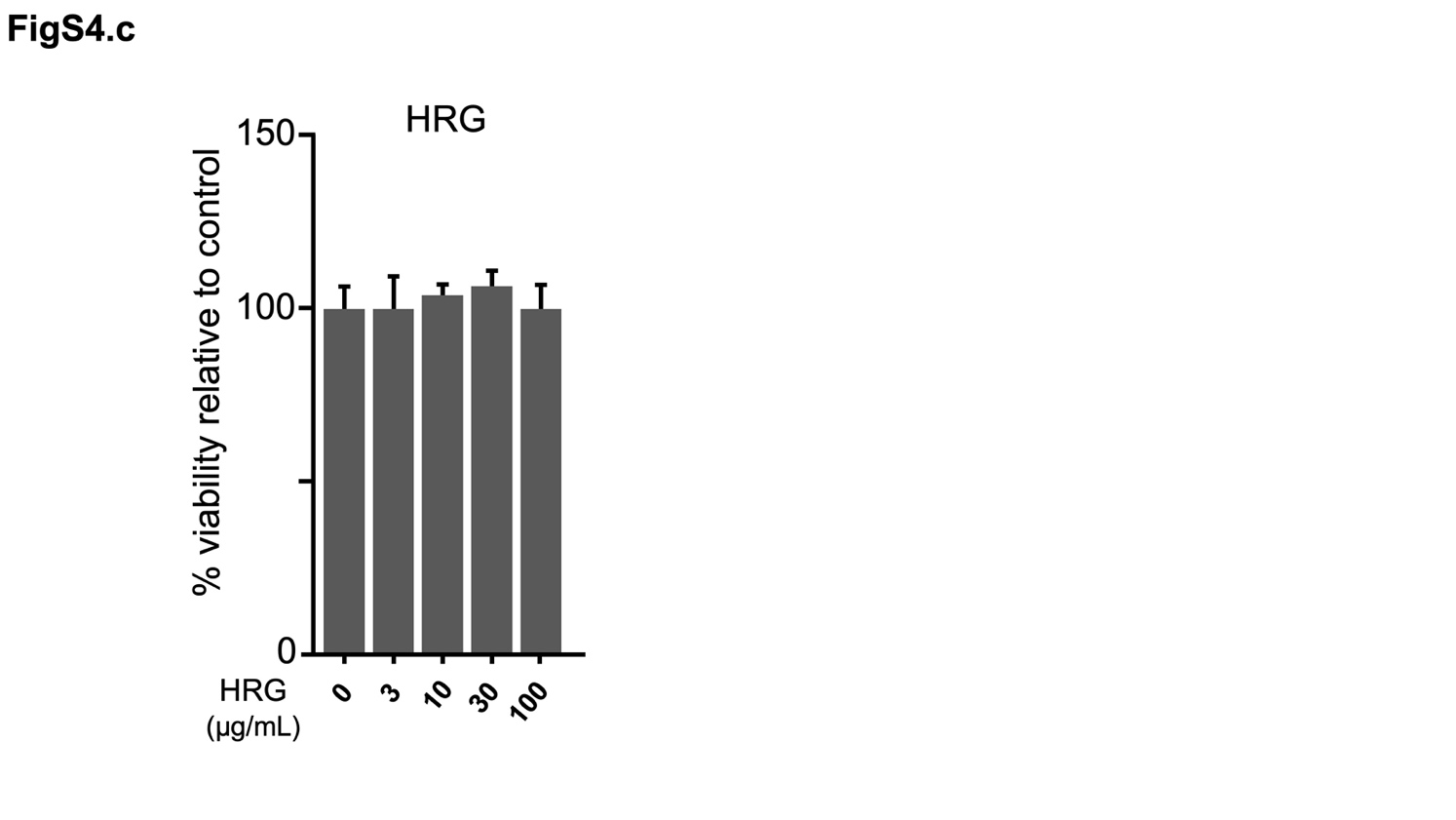

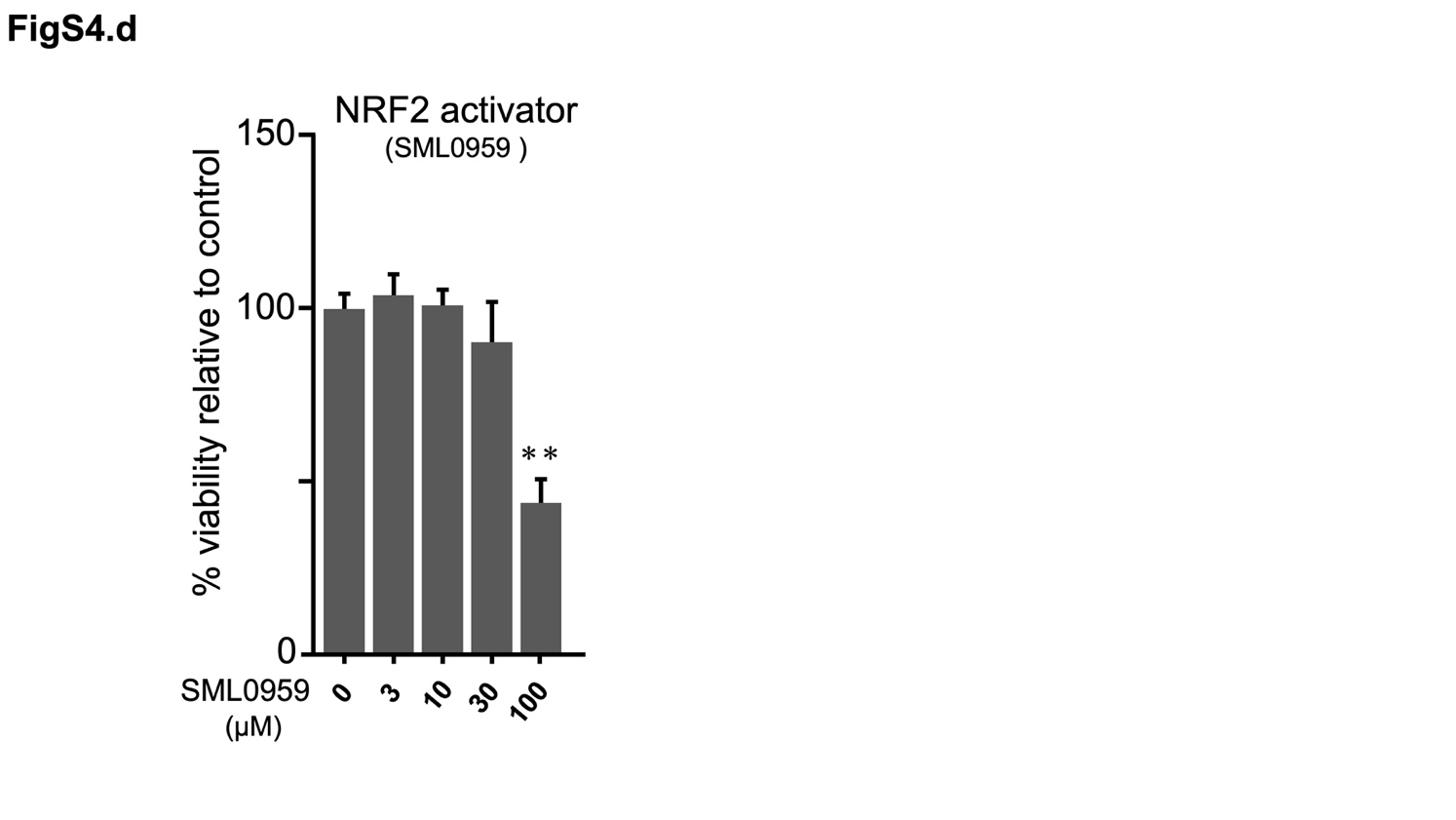
**

**Supplementary Figure 4.** **Effects of TNF-α, HRG, NRF2 activator, and siRNA transfection on the viability of EA.hy926 cells.**

**(A)** Effect of siRNA transfection on cell viability: EA.hy926 cells were transfected with αV or β8 siRNA for 24 h, 48 h, or 72 h.
**(B)** Effect of TNF-α on cell viability: EA.hy926 cells were treated with varying concentrations of TNF-α (0, 3, 10, 30, 100, and 300 ng/mL) for 24 h.
**(C)** Effect of HRG on cell viability: EA.hy926 cells were treated with varying concentrations of HRG (0, 3, 10, 30, and 100 μg/mL) for 24 h.
**(D)** Effect of NRF2 activator (SML0959) on cell viability: EA.hy926 cells were treated with varying concentrations of the NRF2 activator SML0959 (0, 1, 3, 10, 30, and 100 μM) for 24 h. Cell viability was assessed using the CCK-8 assay, and absorbance at 450 nm was measured after a 2-hour incubation. Data are presented as mean ± SD of three independent experiments (n = 3). Statistical significance: p < 0.05 (*), p < 0.01 (), ns = not significant** (Tukey’s post-hoc test).
